# Elevated systemic venous pressures as a possible pathology in prepubertal pediatric idiopathic intracranial hypertension

**DOI:** 10.1007/s00381-024-06594-3

**Published:** 2024-09-10

**Authors:** Casper Schwartz Riedel, Nicolas Hernandez Norager, Maria Bertelsen, Ronni Mikkelsen, Marianne Juhler, Torben Skovbo Hansen

**Affiliations:** 1 Department of Neurosurgery, Copenhagen University Hospital, Rigshospitalet, Copenhagen, Denmark; 2 Department of Paediatrics, Aarhus University Hospital, Aarhus, Denmark; 3 Department of Neuroradiology, Aarhus University Hospital, Aarhus, Denmark; 4 Department of Neurosurgery, Aarhus University Hospital, Aarhus, Denmark

**Keywords:** Pediatric idiopathic intracranial hypertension, IIH, Systemic venous pressure, CVP, Intracranial pressure, ICP, Endovascular venography

## Abstract

**Background:**

Pediatric idiopathic intracranial hypertension (IIH) is a rare and challenging condition. As implied by the nomenclature, the etiologies remain unknown, and multiple etiologies are being investigated. In this study, we explored the potential role of increased systemic or cerebral venous pressure in the pathogenesis.

**Method:**

An observational cohort study following the STROBE guidelines, including prepubertal children with clinical symptoms and imaging findings consistent with IIH referred to the neurosurgical department, was conducted. The patients underwent a comprehensive diagnostic protocol, including MRI, continuous intracranial pressure (ICP) monitoring, and endovascular venography with venous pressure measurements.

**Results:**

The study included 11 consecutive patients (six boys and five girls) with an average age of 2.3 years, and an average BMI of 18.4. Among these, one patient was found to have venous stenosis with a gradient; the other 10 patients presented with normal intracranial anatomy. All patients exhibited elevated venous pressures, with an average superior sagittal sinus pressure of 18.9 mmHg, average internal jugular vein pressure of 17.0 mmHg, and average central venous pressure of 15.9 mmHg. Daytime ICP averaged 12.9 mmHg, whereas nighttime ICP averaged 17.2 mmHg with either A- or B-waves in 10 of the 11 patients. Despite pathological ICP, only three patients had papilledema.

**Conclusions:**

All patients had an increased systemic venous pressure, indicating a possible pathological factor for prepubertal IIH. Additionally, our findings show that young children often only partly meet the Friedman criteria due to a lack of papilledema, emphasizing the need for pediatric-specific diagnostic criteria. Further large-scale studies are needed to confirm these findings and to explore the underlying reasons for this increase in venous pressure and potential new treatment avenues.

## Background

Pediatric idiopathic intracranial hypertension (IIH) is defined as elevated intracranial pressure (ICP) without an identifiable cause. IIH is a diagnostic and treatment challenge, particularly in young children and patients without papilledema. Untreated IIH can lead to irreversible visual impairment, developmental delay, and irreversible cognitive impairment [1–3]. Robust research on this matter is challenging because of the rarity of the disease. The prevalence in Denmark is unknown; however, in the UK, the incidence is 0.17/100,000 in the age group 1–6 years and 0.75/100,000 in the age group 7–11 years [4, 5]. The limited and suboptimal treatment options are closely related to the unidentified underlying pathophysiology and causality [6–10]. Identifying these factors is crucial for transitioning patients from intracranial hypertension of unknown cause to intracranial hypertension secondary to identifiable and potentially treatable causes.

In adults, IIH is associated with risk factors such as female sex and obesity, and a similar pattern is observed in adolescents [11–13]. However, in prepubertal children, these associations are usually not found [11]. Venous outflow obstruction has been proposed to cause IIH in adults [2, 8, 14, 15], hypothesizing that obesity results in increased central venous pressure (CVP) and decreased cerebral venous outflow, leading to increased ICP [16, 17]. Case reports in children of increased intracranial venous pressure due to venous obstruction exist, but the relationship between elevated CVP and elevated ICP of “unknown cause”/IIH in children is unexplored [1, 11, 18, 19].

This study investigated the potential role of elevated venous pressure in prepubertal children with high ICP without hydrocephalus or any other mass lesions on imaging studies. We hypothesized that elevated venous pressure, cerebral or systemic, could contribute to the pathophysiology in some patients by leading to increased intracranial blood volume and, thereby, elevation of ICP. Although elevated venous pressure is unlikely to be the sole mechanism underlying prepubertal pediatric IIH, it might elucidate the etiology in a subset of the patient population.

## Methods

### Study population

This retrospective observational study followed the STROBE guidelines for cohort analyses. The patients were referred to the Neurosurgical Departments at Aarhus and Copenhagen University Hospitals, Denmark, between 2015 and 2023, with behavioral and clinical symptoms strongly suggestive of intracranial hypertension and normal brain imaging. All included patients underwent extensive evaluation prior to referral without a definitive diagnosis and had an inadequate effect of standard care in the pediatric department with persistent severe symptoms, which led to ICP monitoring, confirming increased ICP.

### Diagnostic protocol

The patients underwent a standard diagnostic protocol for children presenting a clinical picture consistent with IIH, including assessments by a neuropediatrician and ophthalmologist with fundoscopy and optical coherence tomography (OCT), continuous day- and nighttime ICP monitoring, and supplementary brain MRI imaging with venograms. As these examinations did not reveal causes to aim treatment at, endovascular arteriography and venography, including venous pressure measurements, were performed as the next step to investigate any venous pathology.

Endovascular venography measurements were obtained using a femoral vein approach under general and local anesthesia at the incision site. Venous pressures were measured in the cerebral venous sinuses, including the superior sagittal sinus (SSS), transverse sinus (TS), sigmoid sinus (SS), and IJV, and in the right atrium (for CVP measurement). In patients with venous stenosis, the venous pressure was measured across the stenosis. For continuous ICP monitoring, measurements were collected using parenchymal ICP sensors (Raumedic Neurovent P ®). Descriptive statistics were used to summarize the variables. Continuous variables are presented as means with standard deviations (SD).

## Results

### Patient cohort

We included 11 consecutive pediatric patients with IIH (six boys and five girls), average age 2.3 years (SD 1.8, range 0.7–6.1), with an average BMI of 18.5 (SD 2.0, range 16.1–23.7). The patients presented with symptoms suggestive of elevated ICP, including nocturnal crying/screaming spells, poor thriving, developmental delay, headaches, and nausea with vomiting episodes. Clinical evaluations revealed papilledema (three patients), headaches (seven patients), nocturnal and recumbent position exacerbations (nine patients), and increased head circumference (six patients) (Table 1).

**Table 1:** Summary of patient demographics and symptoms

Patient	Age (years)	Sex	BMI	Papilledema	Headache	Increasing head circumference	Symptoms worse at night or horizontal	Nausea or vomiting
1	1.4	Male	23.7	Yes	Yes	Yes	Yes	No
2	4.2	Female	18.1	Yes	Yes	Yes	Yes	No
3	1.3	Male	16.9	No	Yes	No	Yes	Yes
4	4.4	Male	20.4	No	No	Yes	Yes	Yes
5	6.1	Female	17.8	No	No	Yes	No	No
6	0.8	Male	18.6	No	Yes	Yes	Yes	Yes
7	1.5	Male	16.1	No	Yes	No	Yes	Yes
8	0.8	Female	17.5	No	Yes	Yes	No	Yes
9	4.1	Female	17.7	Yes	No	No	Yes	No
10	1.2	Male	18.1	No	No	No	Yes	Yes
11	1.8	Female	19.0	No (wide ONSD)	No	No	Yes	No

*BMI* body mass index, *ONSD* optic nerve sheath diameter

All patients complied with the revised Friedman criteria allowing the absence of papiledema, but including the absence of mass lesions and hydrocephalus and documented elevation of ICP, with the modification that cerebrospinal fluid (CSF) analysis was not available, as ICP was measured in the brain parenchyma. However, none of the children had a history or clinical presentation suggestive of meningeal or other CNS diseases, resulting in an abnormal CSF composition.

Two patients had venous stenosis; however, only one had clinically relevant stenosis with a pressure gradient of > 5 mmHg and collaterals, whereas the other had no gradient (Table 2).

**Table 2:** Summary of clinical findings

Patient	Venous stenosis/other	BP (mmHg)	CVP (mmHg)	IJVP (mmHg)	SSSP (mmHg)	Average ICP day (mmHg)	Average ICP night (mmHg)	A- or B-waves
1	No	80/40	22	22	23	n.a. (shunt)	n.a. (shunt)	Yes
2	No	85/45	18	18	18	n.a. (shunt)	15 (shunt)	Yes
3	No	95/45	24	24	24	7.5 (shunt)	7.5 (shunt)	Yes
4	Yes (no gradient)	85/40	16	16	15	14.1	16.2	No
5	No	80/30	19	19	22	17.6	19.8	Yes
6	No	85/30	14	17	21	14.8	17.3	Yes
7	No	80/40	17	n.a	21	10.0	20.0	Yes
8	Yes (gradient of ≥ 5)	80/40	9	13	21	5.1	12.5	Yes
9	No	80/35	n.a	11	13	15.9	16.2	Yes
10	No	85/40	13	16	17	12.6	15.0	Yes
11	No	75/35	14	14	16	12.1	20.2	Yes

*BP* blood pressure on angiogram (systolic/diastolic), *CVP* central venous pressure, *IJVP* internal jugular vein pressure, *SSSP* superior sagittal sinus pressure, *ICP* intracranial pressure, *shunt* ventriculoperitoneal (VP) shunt at the time of venous pressure measurements

### Venous pressure

All patients had elevated venous pressure (Table 2), with an average SSSP of 18.9 mmHg (SD 3.7), IJVP of 17.0 mmHg (SD 4.0), and CVP of 15.9 mmHg (SD 4.8). Notably, venous pressure and ICP were nearly identical in most cases. Prior to venous pressure measurements, the first three patients were equipped with a ventriculoperitoneal (VP) shunt. These three patients still exhibited abnormally high venous pressure with minimal or absent venous pressure gradients (Table 2). A notable case involved a patient whose CVP increased from 9 to 18 mmHg over 40 months, coinciding with the worsening of symptoms (patient 2).

### Intracranial pressure

**Fig. 1:**
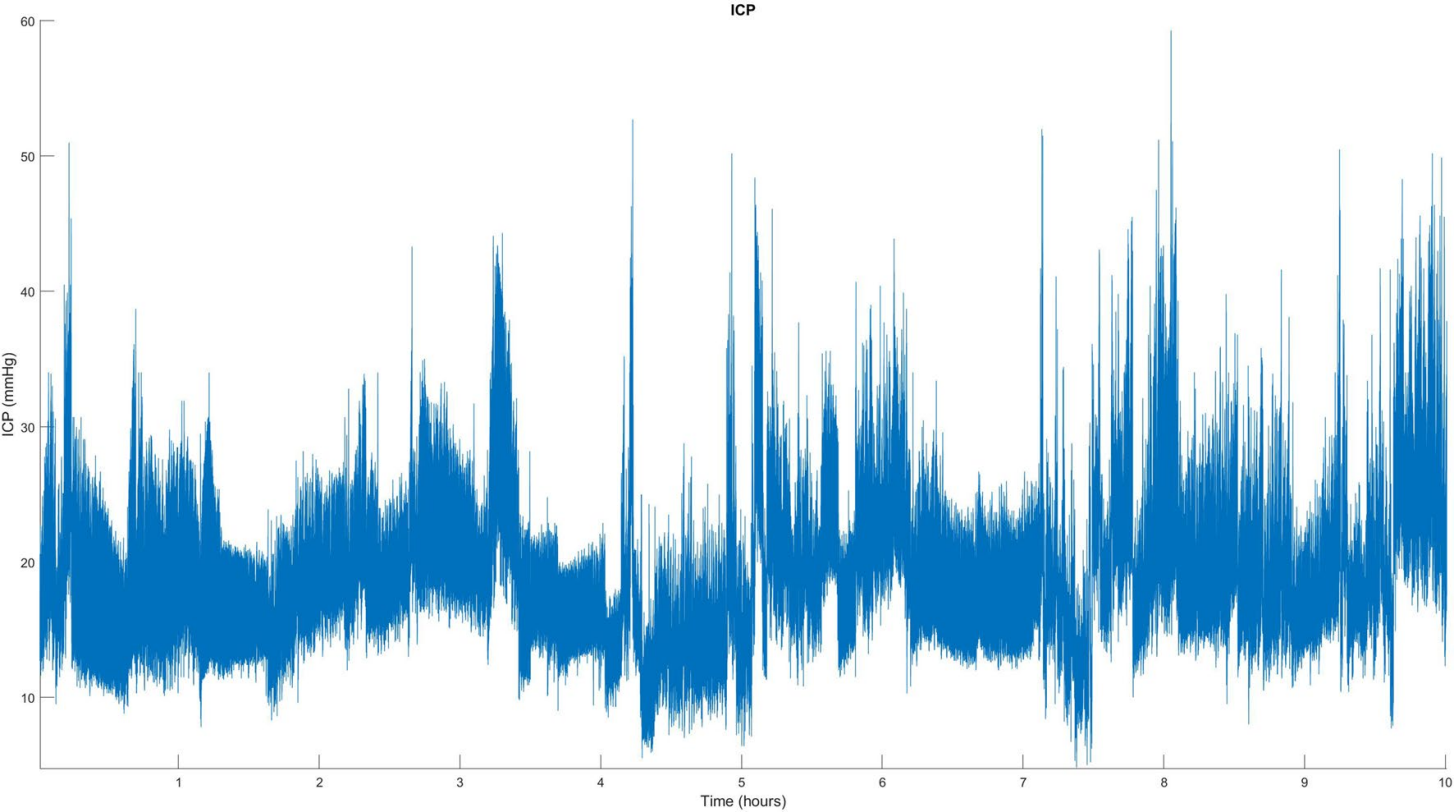
A continuous parenchymal ICP measurement during the night in patient 5, displaying pathologically high ICP with both A- and B-waves. A-waves, also known as plateau waves, display high ICP for a prolonged time, whereas B-waves are short, with repeated elevations of ICP (10–20 mmHg) with a frequency of 0.5–2 waves/min

During the day, the average ICP was 12.2 mmHg (SD 4.0), which increased to 16.1 mmHg (SD 4.1) at night. All patients clearly had abnormally high ICP at night, with Lundberg A- or B-waves detected in 10 of 11 patients with ICP values often exceeding 30–40 mmHg. Transient ICP elevations, such as A- and B-waves, are considered signs of reduced intracranial compliance, and are used to guide decisions for invasive ICP management. Figure 1 illustrates the A- and B-waves in a single patient. Patients 1 and 3 also exhibited similar ICP values; however, continuous ICP values were unavailable for these two patients. In one of these cases (patient 3), we found the highest recorded venous pressure, 24.0 mmHg, in both the SSS and CVP, but near-normal ICP levels at 7.5 mmHg with a VP shunt inserted. Patient characteristics and findings are detailed in Table 2.

## Discussion

To our knowledge, this is the first case series to measure the venous pressure in prepubertal children with IIH. We found consistent pressure elevations in the SSS, IJV, and CVP in all 11 patients. Thus, our findings suggest that increased systemic or intracranial venous pressure may be an important pathophysiological mechanism in patients with prepubertal pediatric IIH. These findings underscore the necessity of acknowledging the dynamic aspects of blood flow and constraints on venous outflow as critical factors in regulating ICP beyond the traditional focus on arterial inflow and direct ICP management [20].

### Elevated venous pressures

Normal CVP ranges from 2 to 7 mmHg, [21] and our cohort displayed pressures ranging from 9 to 24 mmHg, with most patients exhibiting a CVP above 13 mmHg. SSSP and IJVP are generally slightly higher, resulting in a gradient that promotes efficient cerebral venous outflow. The results from our study align with this pattern, finding a gradient of decreasing venous pressure as they approach the right atrium in all patients without a VP shunt or venous stenosis.

The etiology of IIH remains incompletely understood, with various hypotheses proposed, ranging from venous outflow obstruction to metabolic and endocrinological causes [1, 9, 13, 14, 19]. Our study contributes to this ongoing discussion by investigating the role of venous pressure, thereby indirectly leading to venous outflow limitation. Theoretically, a higher CVP would demand increased pressure in the SSSP and IJVP to ensure adequate cerebral venous outflow and drainage, because if the CVP is higher than the pressure in the SSSP and IJVP, the venous blood is not able to flow towards the right atrium. Consequently, venous congestion leads to an increase in intracranial blood volume. The Monroe-Kellie doctrine dictates that increasing venous blood volume requires compensatory volume reduction in the CSF, arterial blood, or brain parenchyma. When compensatory mechanisms are exhausted, any additional increase in intracranial volume results in elevation of ICP [20]. By this mechanism, increased CVP can ultimately result in increased ICP. Conversely, alterations in IJVP or SSSP, independent of changes in CVP, can also result in increased ICP via the mechanism specified above [22]. In our cohort, simultaneous elevation in CVP, IJVP, and SSSP suggests a systemic rather than an intracranial cause for the increased ICP in most patients. However, there are exceptions to this pattern. One patient had venous stenosis (patient 8), primarily raising the venous pressure in SSSP and not CVP to the same extent; this patient had only moderately elevated CVP (9 mmHg) compared to the rest of the cohort, with much higher levels of CVP (13–24 mmHg).

The first three patients were equipped with a VP shunt during venous pressure measurements, exhibiting minimal or absent venous pressure gradients. Patient 3 was of additional interest because of highly elevated venous pressures but only slightly increased ICP. The most obvious interpretation of this finding is that this patient had increased CVP and ICP as the rest of the cohort but that the placement of the VP shunt ameliorated the ICP. However, the VP shunt did not address the underlying venous hypertension. Owing to the absence of pre-shunt venous pressure data, it remains speculative whether the venous pressure gradients were inherently absent or mitigated by the VP shunt, or whether the venous hypertension observed was present before shunt placement, underscoring a limitation in our understanding of the VP shunt effect on cerebral hemodynamics. Notably, if the VP shunt eliminates the venous pressure gradient, it might significantly disrupt normal cerebral blood flow by impeding venous outflow and possibly elevating SSSP even more.

In a review by Sinclair et al., recent evidence suggests that IIH might be viewed as a systemic metabolic disease, extending beyond the commonly affected demographic of overweight females [13]. The review presents a distinctive androgen signature and glucocorticoid dysregulation, and correlates IIH with systemic metabolic diseases, including insulin resistance, type 2 diabetes, and cardiovascular disease [1, 8, 14, 15]. Consistent with established knowledge, the review confirms the association between IIH in adults and truncal fat mass. It is hypothesized that obesity, particularly centripetal fat, results in increased CVP and decreased cerebral venous outflow, leading to increased ICP [13, 16, 23, 24]. Obesity may increase CVP through mechanisms such as increased intra-abdominal pressure, increased intrathoracic pressure, obesity-related cardiomyopathy, and sleep apnea [22]. Notably, sleep apnea causes transient elevations in ICP through intrathoracic pressure changes, highlighting the importance of thoracic hemodynamics in ICP regulation [25, 26]. Studies on animal models and individuals with head trauma have shown that elevated intrathoracic pressure can lead to increased CVP, thereby increasing ICP [27–29]. However, none of the patients in our study was overweight, and all had BMIs within normal ranges, which is typically seen in prepubertal children with IIH [3, 18, 19, 24]. Additionally, the sex distribution was nearly equal, with one more boy than girl, contrasting with the female dominance seen in the adult IIH population. This demographic difference questions whether pediatric and adult IIH share the same underlying pathophysiological mechanisms. Therefore, while increased venous pressure was observed in our cohort, the specific underlying mechanism for this venous hypertension remains unclear, highlighting a gap in the understanding of the pathophysiology of IIH in non-obese populations. A potential explanation for this observation could be the constriction of venous vessels, which may be attributed to heightened venous tone. In pediatric cases, increased intracranial venous pressure due to venous obstruction has been documented; however, the relationship between elevated CVP and pediatric IIH remains unexplored. Although elevated venous pressure is unlikely to be the sole mechanism underlying IIH, it might elucidate the etiology in a subset of patients.

### Clinical implications and future perspectives

In our study, we found that young children often only partly fulfilled the Friedman criteria due to a lack of papilledema, highlighting the need for pediatric-specific diagnostic criteria. Given the strong association between increased venous pressure and ICP, measuring these parameters could be beneficial, especially for patients with suboptimal responses to standard care. More extensive studies are needed to determine whether elevated systemic venous pressure is common in pediatric patients with IIH. If confirmed, this could lead to a paradigm shift in understanding and managing this disease.

In our cohort, acetazolamide (Diamox) was administered to all patients as a standard treatment following adult protocols after IIH diagnosis based on invasive ICP and venous measurements. Patients 1, 3, 7, and 8 also received furosemide, which proved effective. While acetazolamide and furosemide are known to reduce systemic pressure, the primary mechanism by which acetazolamide affects systemic venous pressure and its impact are not yet fully understood and should be clarified in future studies. Future studies should investigate the effects of elevated systemic venous pressure treatment using sildenafil or nitroglycerin patches. Theoretically, these treatments could reduce venous pressure and lower ICP due to their vasodilatory effects, especially in cases where traditional treatments are inadequate.

Our study raises the general question of the role of venous pressure as a regulator of ICP, and its contribution to intracranial hypertension. Investigations of venous pressures across other patient cohorts with elevated ICP could be interesting to determine whether a new therapeutic avenue for ICP regulation exists.

## Limitations

While our study provides valuable insights into pediatric IIH physiology, particularly the relationship between increased venous pressure and pediatric IIH, it has limitations. The primary limitation of this study was its small sample size. However, large sample sizes are challenging because of the low prevalence of the disease. Due to uncertainties in lumbar CSF opening pressure measurements under general anesthesia, we preferred 24-h ICP monitoring while awake and included only pediatric patients with a high suspicion of IIH referred to the neurosurgical department for these measurements. Consequently, the generalizability of our results to the entire pediatric IIH population, particularly those with milder forms, remains uncertain. Despite these limitations, the elevation of venous pressure across all included patients suggests that pathological venous pressure might be a common denominator in pediatric IIH, warranting further investigation in larger cohorts and milder cases of pediatric IIH.

Another limitation is that CVP was monitored in patients undergoing anesthesia. Anesthesia affects the hemodynamic profile, potentially lowering CVP by causing vasodilation and reducing sympathetic tone [30]. Thus, the reported venous pressure may underestimate the actual CVP, reinforcing that the cohort has elevated venous pressures.

## Conclusion

Our study suggests that elevated systemic venous pressure may play a significant role in the etiology of prepubertal pediatric IIH and possibly in some adult cases. Comprehensive studies are needed to confirm these findings and further investigate the relationship between elevated central venous pressure and pediatric IIH. Understanding this connection could significantly improve IIH management and potentially reduce the need for surgical intervention. Additionally, our findings revealed that young children often only partly meet the Friedman criteria owing to a lack of papilledema, underscoring the need for pediatric-specific diagnostic criteria.

## Data Availability

All data supporting the findings of this study are available within the paper. Additional data that support the findings of this study are not openly available due to reasons of sensitivity and are available from the corresponding author upon reasonable request.
